# The level of performance stabilization influences motor adaptation on an isometric force control task

**DOI:** 10.1371/journal.pone.0185939

**Published:** 2017-10-26

**Authors:** Suziane Peixoto dos Santos, Rodolfo N. Benda, Crislaine Rangel Couto, Carlos Eduardo Campos, André Gustavo Pereira Andrade, Guilherme M. Lage, Herbert Ugrinowitsch

**Affiliations:** 1 Universidade Federal do Triângulo Mineiro, Uberaba, Minas Gerais, Brazil; 2 Universidade Federal de Minas Gerais, Belo Horizonte, Minas Gerais, Brazil; 3 Centro Universitário Izabela Hendrix, Belo Horizonte, Minas Gerais, Brazil; 4 Universidade de Itaúna, Itaúna, Minas Gerais, Brazil; Waseda University, JAPAN

## Abstract

This study compared the effects of two levels of performance stabilization on the adaptation to unpredictable perturbations in an isometric control force task with the goal of controlling 40% of the maximum force. The experiment consisted of pre-exposure and exposure phases. In the pre-exposure two levels of performance stabilization were manipulated: a stabilization group (SG) performed three trials in a row while maintaining 40% of the maximum force for three seconds and an absolute error less than or equal to 5% (i.e., the criteria of performance), and a superstabilization group (SSG) performed six blocks of the same criteria. During the exposure phase, the task was the same as that in the pre-exposure phase; however, it was inserted 9 trials of perturbations when the task goal changed to 60% of the maximum force. We measured the %RMSE, RMS from the biceps and triceps brachii and co-contraction. In the pre-exposure phase, both groups showed similar performance and muscle activity. When exposed to the perturbations, SSG performance more quickly returned to the previous level of accuracy, showed lower muscle activation and demonstrated a greater muscle co-contraction than did SG. The results give support to the adaptive process model on motor learning.

## Introduction

Many sports require players interacting with a dynamic environment as well as their ability to reorganize planned actions, especially when the environmental modifications occur in a unpredictable way. This reorganization has been named adaptation, which is characterized by the players’ ability to maintain the performance even facing with environmental changes, i.e. perturbations [1].

Experimentally, adaptation is tested when the researcher introduces a perturbation and observes its effects [2,3] on motor performance and/or on the mechanisms of motor skill control [4,5]. For instance, in the study by Rand et al. [6] the increment in the weight of an object to be transported deteriorated performance adaptation.

An important point here is that adaptation requires not only perturbation, but also the task had already been learned [7,8]. On this concern, recent studies have shown that the higher the level of performance stabilization in the prior acquisition, the better the adaptation [8,9]. In this context, performance stabilization has been defined as the behavior that fluctuates in a narrow bandwidth of error. For example, three levels of performance stabilization have been tested in the context of complex coincident tasks: (1) practice stopping before reaching performance stabilization (i.e., prestabilization); (2) practice up to achievie the performance stabilization (i.e., stabilization); and, (3) practice beyond performance stabilization (i.e., superstabilization). The performance stabilization was operationally identified when a criterion of performance was reached three times in a row. The performance superstabilization had to repeat the same criterion for several times, since motor behavior is non-linear and oscilates between stability and instability [8] and has variability even with learning [10]. Indeed, performance consistency has long been conceived as one of the important characteristics of skillful behavior [11].

The foregoing studies have adopted a complex coincident timing task that shows smaller reorganization of the components of the task for the superstabilization group than the stabilization group [8,9]. Probably it has occurred because the first group has better neuromuscular coordination than the latter group. However, a complex coincident task makes it nearly impossible to measure muscle electrical activity for understanding the differences in neuromuscular coordination. In terms of adaptation, this kind of measure would provide information about how reorganization would took place which could helps to understanding the motor control during adaptation. Based on this, we are pourposing an isometric force control task.

Adaptation to an isometric force control task has been investigated using RMS and co-contraction measures [12,13]. The RMS measure has been used to identify muscle activation patterns associated with muscle strengh [12], and co-activation to identify the quality of motor coordination [14]. This is because the high co-contraction represents a possible increment in joint stiffness [15] as well as the degree of articular stability [13]. The increment of co-contraction can contribute to increasing articular stiffness, which may maintain stability and reduce the burden of a perturbation, therefore helping to reproduce accurate and coordinated movements [16]. Because stabilization and superstabilization levels allow performing similarly under control conditions but differently when facing perturbations, RMS and co-contraction can be tools for understanding the neuromuscular control in different stabilization levels as well as its effects on motor adaptation.

Therefore, the aim of this study was to investigate the effects of two levels of performance stabilization on the adaptation to unpredictable perturbations on an isometric force control task. Understanding muscle control during adaptation can explain the differences on performance between superstabilization and stabilization. Three hypotheses were tested. The first hypothesis was that the levels of stabilization and superstabilization of performance will have similar performances (i.e., Root Mean Square Error—RMSE and muscle activation, i.e., Root Mean Square—RMS) and co-contraction during the pre-exposure (i.e., learning) phase; the second hypothesis was that the performance superstabilization achieved in the pre-exposure phase will lead to a higher performance accuracy (i.e., lower error) and lower muscular activation when compared with the stabilization level in the exposure phase. The third hypothesis was that the superstabilization level achieved in the pre-exposure phase will produce higher co-contraction due to the greater degree of stability required in situations with perturbations.

## Method

### Sample

Eighteen male college students aged 20 to 30 years (mean 22.01 ± 3.07), who had no prior experience with the task participated in this experiment. All participants were self-reported as right-handed and had normal or corrected-to-normal vision. In addition, to confirm the manual preference for the right side (right handed subjects), the Edinburgh Handedness Inventory (Oldfield, 1971) was applied (mean 82.4 ± 3.7). The study was performed in accordance with the ethical standards established in the 1964 Declaration of Helsinki, amended in 1989 and this research was approved by the Local Ethic Committee, in Federal University of Minas Gerais N. 270.382. The informed consente was obtained in writing by all participants and was duly filed.

### Instruments and procedures

The instrument consisted of a tension and compression load cell (type s, *tedea-huntleigh europe limited load cell test data sheet*) attached to an iron plate and connected to an analog-to-digital (A/D) converter (Biovision, Germany) with an input range from -5 to +5 volts, which was connected to a computer with DasyLab software for data acquisition and storage. A plate of iron in the "L" shape was screwed into a wall to hold the tension and compression cell, and support for the right arm was provided with a sideboard to restrict the voluntary extension of the elbow. The experiment started with the standardization of the body position and a height chair adjustment. The volunteers were seated facing the tension and compression cell. The volunteers then placed their arm and shoulder on the iron plate and flexed at 90°, conferred with a goniometer. After standardization, the volunteers’ trunk, shoulders and legs were wrapped by seatbelts to stabilize the position and constrain compensatory movements. A palmar bracing was used to prevent movement of the wrist, so the task was performed only with elbow flexion. The maximum isometric force of the right elbow flexor muscles of each volunteer was determined by flexion with maximum force against the load cell in three trials. Each trial lasted five seconds with five minute intervals between them, and the highest value measured in these three trials was considered the ultimate force; the different percentages of maximum force were adopted in each phase of the experiment.

The volunteers were randomly designated into two groups: the stabilization group (SG) and the superstabilization group (SSG). The difference between the two groups will be explained later.

The EMG signals were collected with active surface electrodes (Silver/Silver Chloride—Midi-Trace® 200 Foam, Graphic Controls Corporation—Canada) with amplifiers (up to 5000 times) in the bipolar configuration. The bipolar circular electrodes of 10mm were positioned on the right side with inter electrode distande of 20mm. About the biceps brachii the electrodes were placed on the line between the medial acromion and the fossa cubit at 1/3 from the fossa cubit, and about the triceps braquii the electrodes were placed at 50% on the line between the posterior crista of the acromion and the olecranon at 2 finger widts medial to the line. The reference electrodes was positioned around the wrist. Before placing the electrodes, sites on the subjects were shaved, and abrasions were removed with fine sandpaper and cleaned with alcohol [17].

After the electrodes were placed and fixed, the electrodos (including the reference electrode) were connected to the equipment and a clinical test was performed to test whether the electrodos have been place properly on the muscle and connected to the equipment so that a reliable signal was recorded. The clinical test guarantees activity of the tested muscle.

### Task

**Fig 1:**
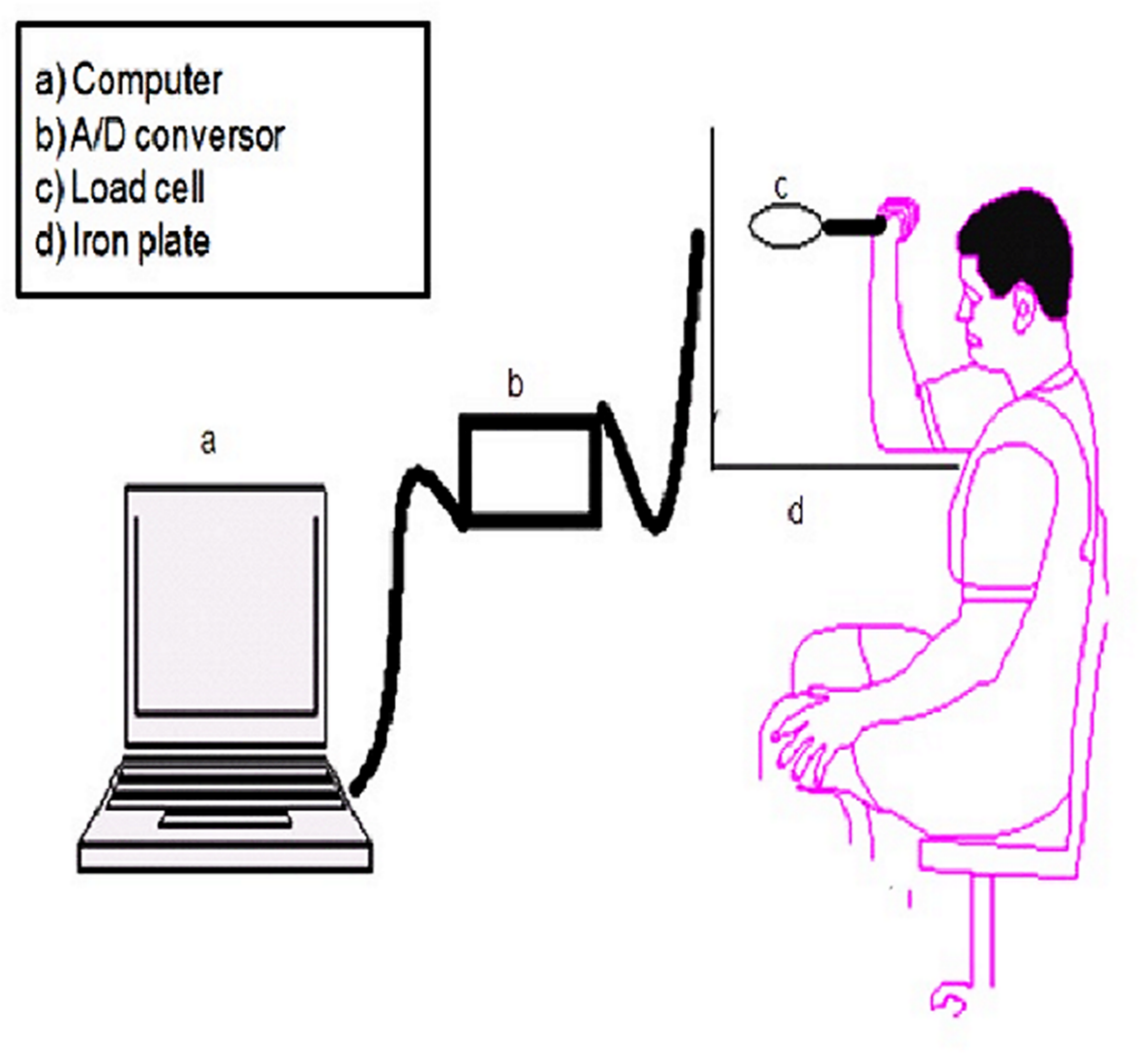
Load cell tension and compression, analog-digital converter and computer.

The task required maintaining isometric force with the goal of controlling 40% of the maximum force of the elbow flexor muscles in the pre-exposure phase and control trials of the exposure phase. For the trials with the perturbation, started with 40% of the maximum force and after 1.5 sec the goal was changed to 60%. In all trials, the time to draw against the load cell was 3 sec. The load cell was connected to a nylon strap with velcro, which was fixed on the palm, allowing the movement of traction (Fig 1).

On the left side of the load cell there was a computer screem with a load/time graphic, with a line that moved from the left to the right on the screen representing the goal to be reached. During the first phase (i.e., pre-exposure phase), the line goal was 40% of the maximum force during the 3 sec. On the exposure phase (i.e., exposure phase), the control trials was the same as the pre-exposure phase, but the perturbation trials had the line goal moved to 60% of the maximum force after 1.5 sec. To both, control and perturbation trials, the goal was to be as accurate as possible on the control of isometric force in relation to the line goal and the total time of the trial was 3 sec, when there was a beep to inform that the trial finished.

### Experiment design

The experiment was divided into two phases: the pre-exposure phase and the exposure phase. To investigate the first hypothesis, during the pre-exposure phase of the experiment, two groups with two levels of performance stabilization were compared: a) a stabilization group (SG) whose participants had to perform three trials in a row maintaining 40% of the maximum force for three seconds with the absolute error less than or equal to 5%. This bandwidth of 5% was found in pilot study, when we found that volunteers did not reach higher accuracy, and b) a superstabilization group (SSG) whose participants had to perform six blocks of three trials in a row maintaining 40% of the maximum force for three seconds with the absolute error less than or equal to 5%. This procedure guaranteed that both groups had different levels of performance stabilization [9] and that the SG and SSG had learned the task [18]. If the effects of the extensive practice on the coincident timing task [9] are similar on a isometric force control task, the differences between SG and SSG will be identified on perturbation trials. On the following day, initially a retention test was performed with the first three trials of the exposure phase to ensure that the task was actually learned. Then, the experiment was conducted with the exposure phase for 117 trials and the subjects continued to practice the same task as that in the pre-exposure phase (i.e., these trials were used as control trials); however, on occasion, the subjects were exposed to 9 pseudo-random trials of perturbations during which the percentage of maximum force was changed to 60%. The percentage of maximum force remained constant during the pre-exposition phase as well as during the control trials in the exposure phase. The unpredictability of the perturbation was guaranteed by the pseudo-random order of the perturbations trials distributed with the 117 control trials [9]. Moreover, this experimental design demanded that the change in planning started when the goal of the task was started to adapt to the imposed perturbations.

### Data analysis

The following dependent variables were used to assess performance: a) %RMSE, a measure to assess performance accuracy since it shows the distance between the force goal and the force performed; b) RMS from the biceps and triceps brachii; and c) co-contraction, a measure to assess the control of movement (i.e., coordination between agonist and antagonist muscles). For the performance variables, after data collection, the raw data were stored and a specific routine was established for the calculation of the dependent variables in the MatLab software (version 7).

The analysis of the EMG signal was also performed with routines developed in the Matlab environment. For signal processing, a butterworth filter with a high-pass of 20 Hz and low-pass of 500Hz was used. The EMG signal was analyzed in the time domain to calculate the RMS values for the analysis of muscle activity during the three-second trials. The pattern of the co-contraction of the arm muscles was analyzed using the following formula, according to Hammond et al. [19]:

Co−Contraction=EMGantagonist*100÷EMGantagonist⊕agonist

### Statistical analyses

For statistical analysis, we used a single average value of RMS and RMSE% from each subject in each condition tested. The pre-exposure data were grouped in three trials; the initial and final phases for the comparison of the two stabilization levels of performance was compared by two-way analysis of variance (Groups 2 x 2 Blocks) because the subjects used distinct amount of practice to achieve the stability measurement [9,20,21]. In the exposure phase, firsty we run a t test comparing the first three trials of SG and SSG as a retention test by a t test. Later, three blocks of nine trials were analyzed and compared by two-way analysis of variance (2 Groups x 3 Blocks): One block with the nine perturbations trials (PERT); one blocked with the nine trials immediately before each perturbation (PRE) and one block with the nine trials immediately after each perturbation (POST). This measure ultimately compares the Pre-Perturbation (PRE), Perturbation (PERT) and Post-Perturbation (POST) trials. Statistical analysis was performed using STATISTICA 8.0 software for the comparisons using ANOVA for repeated measures and Tukey’s post-hoc test for pairwise comparisons and adopting a significance level of p<0.05. The vertical bars on the graphs denote a 95% confidence interval.

## Results

### Pre-exposure phase

**Fig 2:**
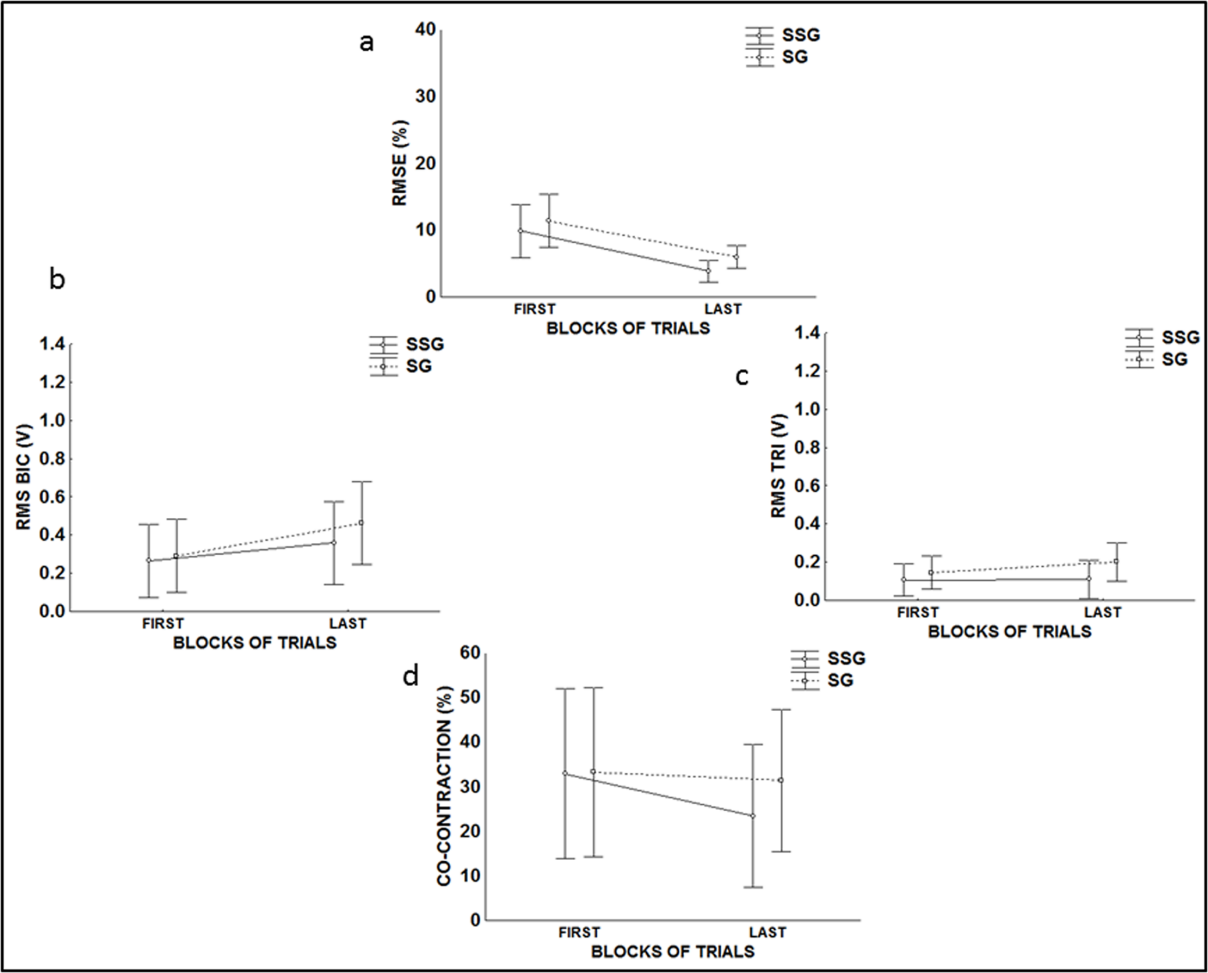
Measures from SG and SSG in the pre-exposure phase. a) Mean of root mean square error (RMSE%); b) (RMS) of biceps; c) (RMS) of triceps; d) Co-contraction.

Fig 2A shows that, even with the two levels of performance stabilization, the performance accuracy (%RMSE) of the two groups was similar (F(1, 6) = 1.48, p = 0.26, *η*_p_^2^ = 0.53), and there was no interaction between the groups and blocks (F(1, 6) = 0.10638, p = 0.75). However, both groups significantly increased the accuracy of their performance from the beginning to the end of this phase (F(1, 6) = 37.05, p = 0.01). Moreover, the SG practiced 51±44.85 trials to reach stabilization and the SSG 179.13±32.41 trials to reach superstabilization, and this difference was significant (p = 0.01, *η*_p_^2^ = 0.69).

Fig 2B shows that the muscle activity of the two groups was similar in the biceps muscle (F(1, 6) = .32, p<0.58, *η*_p_^2^ = 0.37). However, a significant interaction was found (F(1, 6) = 1.3167, p = 0.02), and the post-hoc test detected that SG increased muscle activity throughout this phase (p = 0.05). Fig 2C shows that the two groups had similar muscle activity in the triceps (F(1, 6) = 1.5694, p = 0.25, *η*_p_^2^ = 0.43). However, a significant interaction was found (F(1, 6) = 8.0740, p = 0.02), and the post-hoc test detected that SG increased muscle activity throughout this phase (p = 0.05). Fig 2D shows that the co-activation of both groups was similar (F(1, 6) = .17762, p = 0.68 *η*_p_^2^ = 0.23). Moreover, there was no significant difference between the blocks (F(1, 6) = 5.3090, p = 0.06) or interactions (F(1, 6) = 2.3822, p = 0.17).

**Fig 3:**
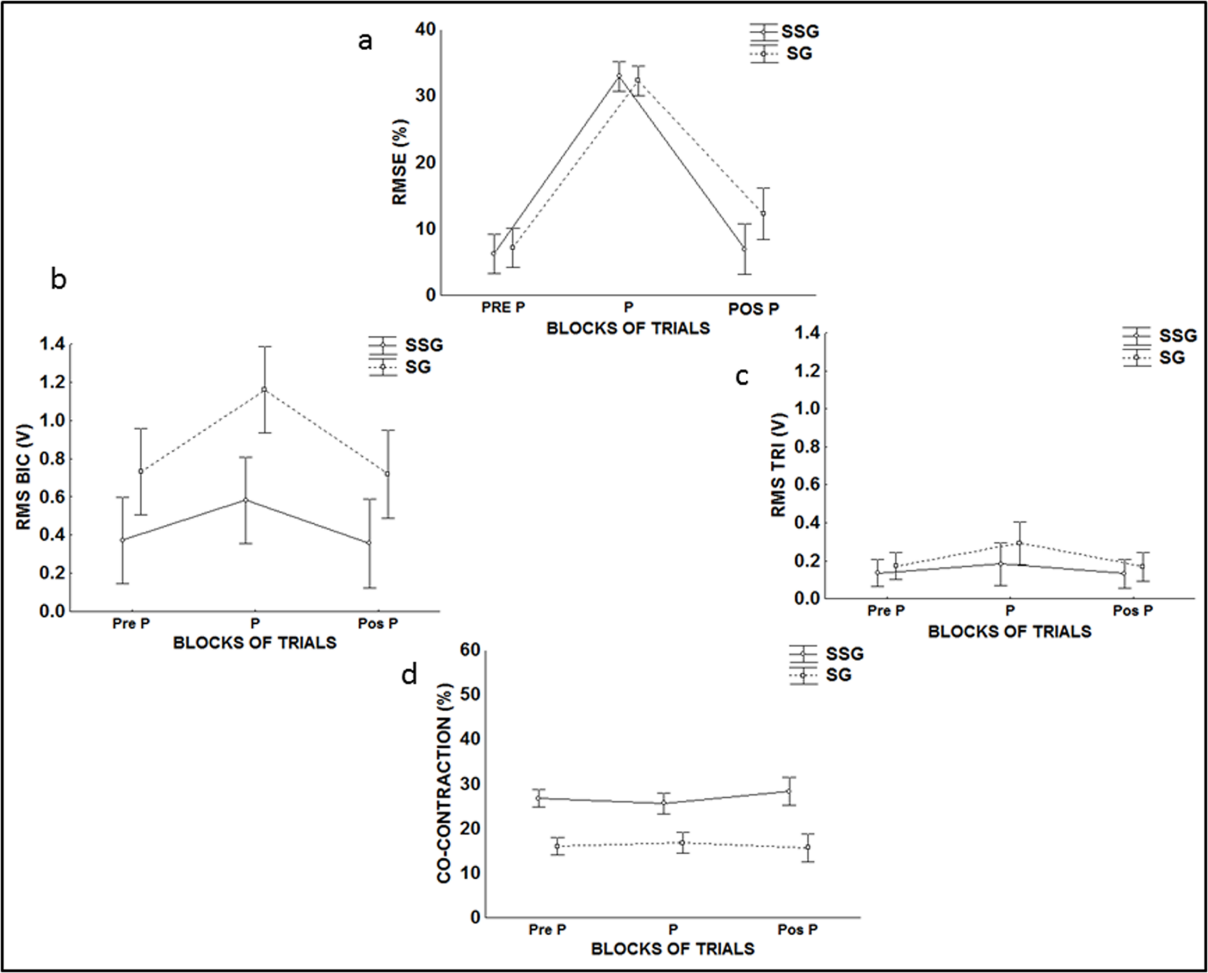
Measures from SG and SSG in exposure phase. a) Mean (RMSE%); b) (RMS) of biceps; c) (RMS) of triceps; d) Co-contraction.

The comparison of the retention test showed that both groups had similar performance (p = 0.68, *η*_p_^2^ = 0.74). Fig 3A shows that, when exposed to perturbations, significant differences between the blocks (F(2, 12) = 434.33, p<0.01, *η*_p_^2^ = 0.53) as well as significant interactions (F(2, 12) = 5.2089, p<0.02) were found. The post-hoc test detected that in PERT, the accuracy of both groups decreased (p = 0.01); however, in POST only the SSG returned to the same level of accuracy as that in PRE (p = 0.99). At last, the groups had similar performances in the PRE, PERT and POST trials (F(1, 6) = 1.7625, p<0.23).

Fig 3B shows that there was a significant interaction (F(2, 12) = 17.012, p<0.01, *η*_p_^2^ = 0.29), and the post-hoc test detected that, in PERT, SSG had lower muscle activity compared with that of SG (p = 0.02). Moreover, muscle activation was significantly lower for SSG compared with that of SG (F(1, 60) = 11.116, p<0.01). Finally, the difference between the blocks in terms of biceps muscle activity (F(2, 12) = 154.19, p<0.01), and the post-hoc test detected that, during PERT, muscle activity increased significantly (p = 0.01).

Fig 3C shows that there was a significant interaction (F(2, 12) = 8.58, p<0.01, *η*_p_^2^ = 0.56), and post-hoc test detected that the muscular activity of the SG increased during PERT when compared with that of during PRE (p = 0.01) and POST (p = 0.01). The SSG did not alter the level of muscle activation in PERT to maintain the same performance. Moreover, a significant difference was found between the blocks (F(2, 12) = 45.183, p<0.01), and the post-hoc test detected that the muscle activation of the triceps increased during PERT (p = 0.01). Finally, no difference between the blocks in terms of triceps muscle activity (F(1, 6) = 1.54, p<0.26).

Fig 3D shows that there was a significant interaction (F(2, 12) = 4.37, p<0.03, *η*_p_^2^ = 0.69). The post hoc test detected that SSG had a higher level of co-contraction than did SG in PRE (p = 0.02), PERT (p = 0.04) and POST (p = 0.01). Moreover, the co-contraction of both groups did not change during PRE, PERT or POST (F(2, 12) = 0.86, p = 0.44). Finally, SG did not change the co-contraction with perturbation (p>0.05).

## Discussion

The aim of this study was to investigate whether performance superstabilization could improve motor adaptation to unpredictable perturbations compared with performance stabilization. Adaptation is considered the capacity or competence to maintain performance when facing perturbations [9], which was assessed through changes in movement activation and muscle activity during task execution [22]. We found that a higher level of performance stabilization was associated with lower muscle activity and higher co-activation during the moments PRE, PERT and POST. These results addressed the aim of this study.

The first hypothesis of this study was that both levels of performance stabilization would have similar performances and muscle electrical activity during the pre-exposure phase. This hypothesis was proposed because, under constant conditions, performance stabilization should be sufficient to produce a good performance [23]. The results showed that both groups diminished RMSE and had similar biceps and triceps RMS and co-activation, although superstabilization practiced more trials than stabilization. This result suggests that the manipulation of both levels of performance stabilization did not have any effect under constant conditions. This result could represent the finite process of motor learning found in the automatized stage of learning [24]. Therefore, the level of stabilization of both groups was sufficient for organizing a similar structure of control of the motor skills, as represented by the RSE and co-activation. The first hypothesis was confirmed.

However, if motor learning is a continuous process that extends beyond performance stabilization [25], we expect that superstabilization would guarantee better performance than stabilization under conditions that require changes in the skills previously learned [26]. This expectation was our second hypothesis.

The exposure to perturbations (PERT trials) deteriorated the performance of both groups, independently of the level of performance stabilization. However, only the superstabilization group returned to the performance level of the PRE trials immediately on POST trials. Since the two levels of stabilization performed similarly on retention test, we can say that stabilization learned to control the 40% of the maximum force as much as superstabilization, but the last had better ability to manage changes in the task. Thus, although superstabilization does not provide full capacity to adapt, it produces a higher ability to return to the performance level observed in control trials, which is consistent with the results of Fonseca et al. [9]. The worse performance during perturbations is caused by the difficulty of changing the planned action after the onset of the movement. If the perturbation is predictable and constant [8], the performance in the perturbation trials can be accurate because it is possible to plan the necessary changes to the control of the skill in advance [27,28].

Studies that have investigated the effects of levels of performance stabilization on adaptation [9] did not analyze neuromuscular coordination measures. Superstabilization led to higher accuracy with lower activity of biceps RMS in PERT, probably as a result of the higher level of performance stabilization in the pre-exposure phase. Although Proteau (1992) showed that extensive practice improves motor learning, it can be influenced by individual differences because all the volunteers have the same amount of practice and can reach different levels of learning inside the same group. The experimental design of our study respects the individual differences as well as the previous experience, since participants can reach the same performance with different amount of practice. Moreover, stabilization practiced until reach the criterion of performance and superstabilization repeated the criterion for many times. This condition provided superstabilization to a different ability to change the control, which has support on the similar performance during retention test.

Previous studies also found an increase in isometric force control associated with reduced muscle activation [29], which can be a result of the increase in intra- and inter-muscular coordination resulting from practice. For example, Gabriel [30] found reduced levels of muscle activation throughout the practices. Beyond the measure of biceps, the evaluation of intra-group behavior of the triceps RMS showed that superstabilization resulted in a more stable behavior in PERT when the subjects maintained similar strength control despite the new task goal, probably as a result of the appropriate level of co-activation. This is due practice beyond stabilization, when variability increases again [14] and the learner can reach more information related to the task. During this process the learner acquires more information related to the task that is used to adapt to perturbation. Different behavior was observed for the stabilization level in response to inappropriate co-activation, which resulted in a difficulty of sustaining contraction or even anormal relation of force and eletromyographic activity [31]. A typical behavior can be reinforced in this group and refers to the decrease in the antagonist activity at the end of the movement because this muscle should contribute to the deceleration of the movement; consequently, an opposite behavior was expected [32].

Variations in eletromyographic activity are observed throughout learning [33,34] that should be related to adaptive changes in the articular torque required to produce necessary compensatory strengh [35]. The RMS behavior of the superstabilization can be related to the changes from learning as well as the adaptation process [36], supporting the proposal that practice beyond performance stabilization can improve the capacity to adapt to perturbations. These results are in accordance with Fonseca et al. [9].

The co-contraction results should be highlighted because we did not find studies investigating the level of performance stabilization that also analyzed muscle co-contraction, which indicates movement control. The third hypothesis of the study was that practice until performance superstabilization should have a higher co-contraction than practice until performance stabilization, which was confirmed. Isometric force is dependent on the agonist and antagonist muscle activation [19]. Co-contraction measurement has been adopted to analyze the quality of coordination [14] as well as the joint stiffness [13]. Increases in co-contraction are necessary for improvements in joint stiffness [37] as well as to diminish task complexity throughout motor learning [38]. For exemple, Benecke et al. [39] interpreted the co-contraction as an explicit away to control the stiffness through a sequence of movements involving pressing a knob and elbow flexing.

The highest superstabilization muscle co-contraction in the PRE, PERT and POST blocks shows higher muscle organization when compared with that of stabilization. For example, Ugrinowitsch et al. [8] found a strong correlation between structure variability before perturbation and a small change in performance when facing perturbation for superstabilization. High variability after performance stabilization can occur and higher muscle co-contraction improves join stiffness [15] and gives higher ability to adapt. Muscle co-contraction contributes to higher muscular stiffness and prepares joints for possible perturbations [40,41]. Moreover, muscle stiffness adjustments can help correction (i.e., adaptation) and minimize perturbation effects on high jumps [42]. During constant practice (i.e., pre-exposure phase), co-contraction was similar in both levels of performance stabilization. However, the exposure phase brought some unpredictable perturbations and the higher co-contraction from superstabilization indicates pre-preparation for adjustments on force control. This pre-preraration did not occur on stabilization level and can be the source of difficulty to adapt, indicating that both levels of performance stabilization use different mechanisms of control. While stabilization level uses predominantly feedback control [43] for corrections to reach the task goal, superstabilization seens to combine pre-programed with feedback control [44,45] (e.g., Brownstone, Bui & Stifani, 2015; Shemmell, Krutky & Perreault, 2010).

The analysis high level of co-contraction data during pre-exposure phase (i.e., constant practice) could be interpreted as high levels of individual differences and problems on data reliability, but when facing perturbation co-contraction variability of both groups diminished. High performance variability during constant practice has been found in previous studies [5,8,10], which diminished with perturbations and the high strucuture variability before perturbation was related to better performance adaptation [8]. Thus, we can say that under constant condition, high co-contraction variability is related to high performance variability and low co-contraction variability under perturbation condition is related to ability to adapt. In this way, higher co-contraction with superstabilization can indicate a more adaptive structure that controls motor skills, which explains the better adaptation from superstabilization found by [8,9]. Practice until performance superstabilization can help the system acquires an abundancy of informations, and abundant systems adapt easily [46] when acquires more information related to the task. This amount of information can increases co-contractions that improves performance when facing perturbations.

In general, studies show that changes in movement require incremental changes in articular stiffness to maintain movement stability. The high demand from articular stability requires a mechanism with continuous and dynamic articular stiffness to guarantee adaptability to environmental demands [46]. Previous studies that investigated adaptation in motor learning showed changes in the temporal organization of movements in complex coincident tasks [9,25] but not in muscle activity. This study provides further information regarding changes in muscle activity and the adaptive process of motor learning.

## Conclusions

Unpredictable perturbations require rapid adjustments in sport activities, specially after the onset of movement. During this experiment, changes in the task goal i.e., increased percentages of maximum isometric force, resulted in increased error measurements during perturbation for both groups, but superstabilization resulted in a higher level of co-contraction even during perturbation and error returned to the pre-perturbation level. These results contribute to the understanding of the adaptive process: practice beyond performance stabilization produces a similar performance to that of practice until performance stabilization but with smaller muscule activity and faster movement reorganization as soon as the perturbation is withdrawn. Summarizing, the results show that practice beyond performance stabilization change neuromuscular control improving performance accuracy during adaptation. Future works should also address the mechanisms of control the movement.

## Supporting information

S1 Fig Individual values and means during the moments PRE, PERT, and POS of biceps, triceps, co-activation and RMSE from SSG and SG groups. (XLSX)
